# Can Residency Programs Detect Artificial Intelligence Use in Personal Statements?

**DOI:** 10.7759/cureus.88969

**Published:** 2025-07-29

**Authors:** Nicole Cumbo, Whitney Williams, Joseph C Canterino, Noelle Aikman, Jonathan D Baum

**Affiliations:** 1 Obstetrics and Gynecology, Hackensack Meridian Jersey Shore University Medical Center, Neptune, USA; 2 Obstetrics and Gynecology, St George's University School of Medicine, St George's, GRD

**Keywords:** artificial intelligence and education, artificial intelligence in medicine, artificial intelligence in scientific writing, electronic residency applications, personal statement

## Abstract

Objective: To evaluate widely used artificial intelligence (AI) detectors’ ability to identify ChatGPT’s (OpenAI, San Francisco, CA, USA) use in personal statements submitted as part of the residency program application.

Materials and methods: This qualitative analysis was performed to evaluate the ability of three different AI detectors to detect the use of AI in personal statements submitted as part of residency applications for obstetrics and gynecology. A total of 25 writings were selected and analyzed by GPTZero (Princeton, NJ, USA), Undetectable AI (Sheridan, WY, USA), and Winston AI (Montreal, Quebec, Canada).

Results: In total, 25 separate writing samples of approximately 700 words were entered into three different AI detectors. AI-generated works had high rates of AI-detection, while classic literature samples had low rates of detection. Human-written personal statements before and after the availability of ChatGPT technology results were mixed, with results ranging from 64-100% and 3-100% of content appearing to be AI, respectively.

Discussion: AI-chatbots have been shown to produce writing that may be indistinguishable from human work and may already be commonly used to create personal statements. It is unclear who is utilizing ChatGPT in their writing, and residency programs everywhere will seek a reliable way to detect unethical usage. This study shows that available AI detectors may be able to detect AI use in applicants’ personal statements, but the use of invalidated tools may harm honest applicants.

Conclusion: Residency programs may be able to detect AI use in personal statements by utilizing AI-detection tools. Clear guidelines regarding the appropriate use of AI and authorship must be developed in order to maintain the integrity of student submissions.

## Introduction

Artificial intelligence (AI) is here, and many are encouraging its use in scientific writing to democratize knowledge, help writers express ideas more effectively, and gain insights from a diverse range of individuals [1]. AI has also gained traction as a writing assistant at both the college and postgraduate levels [1,2]. While the definition of authorship has been well established for decades, the expansion of AI as “co-author” begs the question: Who gets the credit for significant contributions in academic work?

Editorial stances on AI use in scientific writing vary. According to the Electronic Residency Application Service (ERAS) Applicant User Guide, the personal statement “must be your own work and not the work of another author or the product of artificial intelligence” [3]. However, the definition of a product of AI is unclear, as they cite acceptable forms of use are brainstorming or editing the personal statement [3]. In response to concerns regarding the undisclosed use of AI in academics, AI detection software has been developed and promoted as a potential solution.

Personal statements are an integral and required component of the residency program application. The personal statement gives an applicant the unique opportunity to highlight those talents or attributes that set them apart from the rest of the applicant pool. 

While undisclosed AI use in scientific writing can lead to taking credit for work the writer did not create, its use within personal statements can mislead residency programs and misrepresent applicants in an already very competitive process. This academic dishonesty can disrupt the resident selection system.

Studies suggest that AI-generated personal statements may be indistinguishable from original work, and may give applicants an advantage as they have high acceptability to residency programs [4-6]. Universities worldwide are struggling to define the proper use of AI within their institutions. With this ease of access and growing popularity, many institutions now face the same questions: Who is using or should be using AI, and to what degree? Can such usage be detected in a reliable fashion? Can AI usage be regulated, and if not, should disclosure of its use be required in academic or professional situations?

The American Medical Association (AMA) defines authorship via the International Committee of Medical Journal Editors (ICMJE) criteria, including making substantial contributions, drafting and revising, participating in final approval of the work, and being accountable for the work’s accuracy and integrity [7]. Publication of unoriginal material is considered unethical, and the ICMJE recommends acknowledging all non-author contributions, which would include AI. It appears that residency programs may have no choice but to turn to AI detection systems, which are able to analyze a writing sample and output the probability that an entered text was generated by AI.

The purpose of our study is to evaluate three AI detectors’ ability to identify AI use in personal statements. Our secondary goal is to preserve the integrity of the students’ submissions and allow for the judicious use of AI in postgraduate education.

## Materials and methods

This is a qualitative analysis of the ability of three leading AI detection softwares GPTZero (Princeton, NJ, USA), Undetectable AI (Sheridan, WY, USA), and Winston AI (Montreal, Quebec, Canada) to detect the use of AI in personal statements submitted as part of residency application to obstetrics and gynecology [2,8,9]. GPTZero is a popular software released by ChatGPT (OpenAI, San Francisco, CA, USA), one of the most commonly used AI technologies. We chose Undetectable AI and Winston AI since they are highly ranked AI detectors [9].

A total of 25 writing samples of about 700 words each were analyzed in November 2023: (i) Five excerpts from classic novels from the 1980s (*It* by Stephen King, *Handmaid’s Tale* by Margaret Atwood, *Number the Stars* by Lois Lowry, *The Color Purple* by Alice Walker, and *Lonesome Dove* by Larry McMurtry) chosen as a control since we know they were crafted prior to the wake of AI technology; (ii) Five excerpts from classic novels from the 1800s (*Moby Dick* by Herman Melville, *Pride and Prejudice* by Jane Austen, *Jane Eyre* by Charlotte Brontë, *Frankenstein *by Mary Shelley, and *The Adventures of Huckleberry Finn* by Mark Twain) chosen as a control since we know they were crafted prior to the wake of AI technology; (iii) Five AI-generated writings (referred to as "AI-Generated 1-5") created and used in the study as controls since we know the origin of these writings is 100% AI; (iv) Five personal statements submitted to the Electronic Residency Application System (ERAS) for Obstetrics and Gynecology residency for the 2023-2024 application cycle (referred to as "Real Personal Statements 1-5") chosen as they are real data from the application cycle, where students have access to AI technology; (v) Five personal statements submitted to ERAS prior to the launch of ChatGPT in November 2022 (referred to as "Old Personal Statements 1-5") chosen as controls since we know these students did not have access to AI technology (These were a convenient sample from old personal statements of past residents within an obstetrics and gynecology residency program).

Each writing sample was approximately 700 words since that was the average length of the personal statements submitted by real applicants. These cohorts were chosen as they are convenient samples, with 2023-2024 applicants having access to ChatGPT and serving as the test group, and with the applicants prior to ChatGPT with no access to ChatGPT and serving as a control. Excerpts from popular literary works from the 1800s and from the 1980s were chosen as a second control since they were written prior to ChatGPT development.

For the AI-generated submissions, we asked ChatGPT to write a personal statement for a fourth-year medical student applying for obstetrics and gynecology residency and repeated this request five times: “Write a 700-word personal statement for a medical student pursuing obstetrics and gynecology residency who ___”. We used the following prompts to obtain five different personal statements: ‘is underrepresented in medicine’, ‘went on a mission trip to Ghana’, ‘has an interest in Maternal Fetal Medicine’, ‘has an interest in advocacy’, and ‘experienced a personal loss’. Five different prompts were used to show a variety of data to the AI detection software.

Each of these writing samples was then entered individually into GPTZero, Undetectable AI, and Winston AI, the “check origin” action was utilized, and the output was recorded. The output for GPTZero includes the probability that the work is entirely created by AI, a proportion of how many of the sentences it estimates were generated by AI, and if the interpretation is likely human, AI, or a mix. Undetectable AI and Winston AI results included the percentage of content that appeared human. Our analysis was exempt from review by the Institutional Review Board since we did not include any patient-level data.

## Results

In total, 25 separate writing samples of roughly 700 words were entered individually into GPTZero, Undetectable AI, and Winston AI. A summary of results for all writing samples grouped by type for the three AI detectors, Undetectable AI, Winston AI, and GPTZero, is seen in Table 1. 

**Table 1:** Results of AI detectors on all writing samples Table including results for all writing samples grouped by type for the three highly-ranked AI detectors: 1. Undetectable AI, 2. Winston AI, 3. GPTZero [9]. List of 25 writings run through GPTZero: (i) Five personal statements submitted to the Electronic Residency Application System (ERAS) for Obstetrics and Gynecology residency for the 2023-2024 application cycle (Real Personal Statements 1-5); (ii) five personal statements submitted to ERAS prior to the launch of ChatGPT in November 2022 (Old Personal Statements 1-5); (iii) five AI-generated writings (AI-Generated 1-5), (iv) five excerpts from classic novels from 1800s (*Moby Dick* by Herman Melville, *Pride and Prejudice* by Jane Austen, *Jane Eyre *by Charlotte Brontë, *Frankenstein* by Mary Shelley, and *The Adventures of Huckleberry Finn *by Mark Twain); and (v) from the 1980s (*It* by Stephen King, *Handmaid’s Tale* by Margaret Atwood, *Number the Stars* by Lois Lowry, *The Color Purple* by Alice Walker, and *Lonesome Dove* by Larry McMurtry).

Category	Personal statement	GPTZero			Undetectable AI	Winston AI
		Human vs AI	Probability entirely by AI	Sentences likely AI-generated	Content appearance	Content appearance
1800s	Moby Dick	Likely human	0%	0/63	100% human	100% human
	Pride and Prejudice	Likely human	23%	0/80	100% human	100% human
	Jane Eyre	Likely human	0%	0/33	100% human	100% human
	Frankenstein	Likely human	0%	0/30	100% human	99% human
	Huckleberry Finn	Likely human	0%	0/35	100% human	100% human
1980s	It	Likely human	0%	0/49	100% human	100% human
	Handmaid’s Tale	Likely human	0%	0/59	100% human	100% human
	Number the Stars	Likely human	2%	0/67	100% human	100% human
	The Color Purple	Likely human	2%	0/101	100% human	100% human
	Lonesome Dove	Likely human	2%	0/35	100% human	100% human
AI	AI-Generated 1	Likely AI	93%	28/28	0% human	0% human
	AI-Generated 2	Likely AI	93%	30/30	50% human	0% human
	AI-Generated 3	Likely AI	92%	28/28	0% human	0% human
	AI-Generated 4	Likely AI	92%	32/32	0% human	0% human
	AI-Generated 5	Likely AI	93%	27/27	100% human	0% human
Real applicants	Real Personal Statement 1	Likely a mix	48%	24/29	100% human	53% human
	Real Personal Statement 2	Likely a mix	84%	36/36	100% human	95% human
	Real Personal Statement 3	Likely a mix	49%	19/31	100% human	63% human
	Real Personal Statement 4	Likely human	18%	0/28	100% human	100% human
	Real Personal Statement 5	Likely AI	91%	26/26	50% human	3% human
Real applicants prior to launch of ChatGPT	Old Personal Statement 1	Likely a mix	36%	18/49	50% human	100% human
	Old Personal Statement 2	Likely human	30%	0/34	100% human	100% human
	Old Personal Statement 3	Likely a mix	29%	15/43	100% human	100% human
	Old Personal Statement 4	Likely human	25%	0/37	100% human	100% human
	Old Personal Statement 5	Likely human	11%	0/35	100% human	100% human

**Figure 1:**
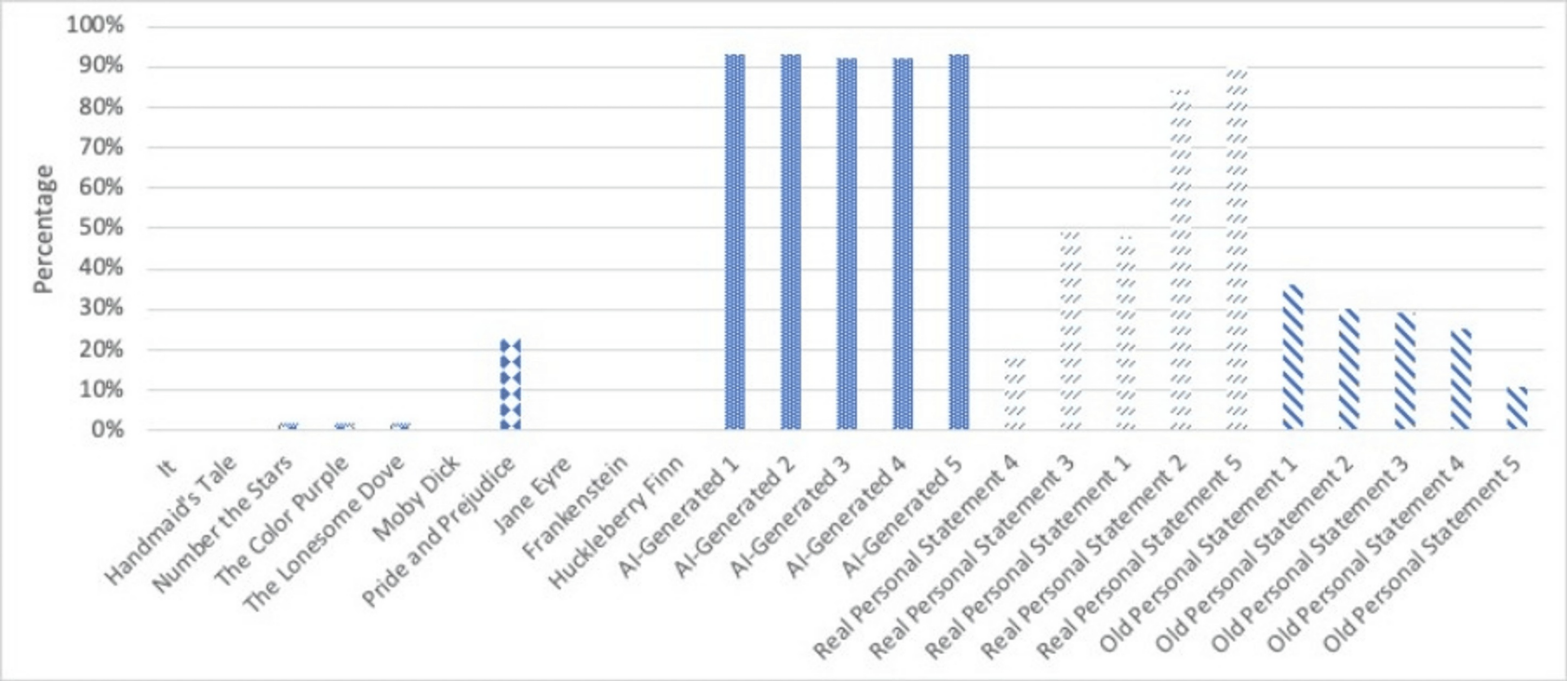
GPTZero results Probability entry entirely by AI based on the output of GPTZero, evaluating the 25 writing samples.

The probability of being written entirely by AI per GPTZero is seen in Figure 1.

**Figure 2:**
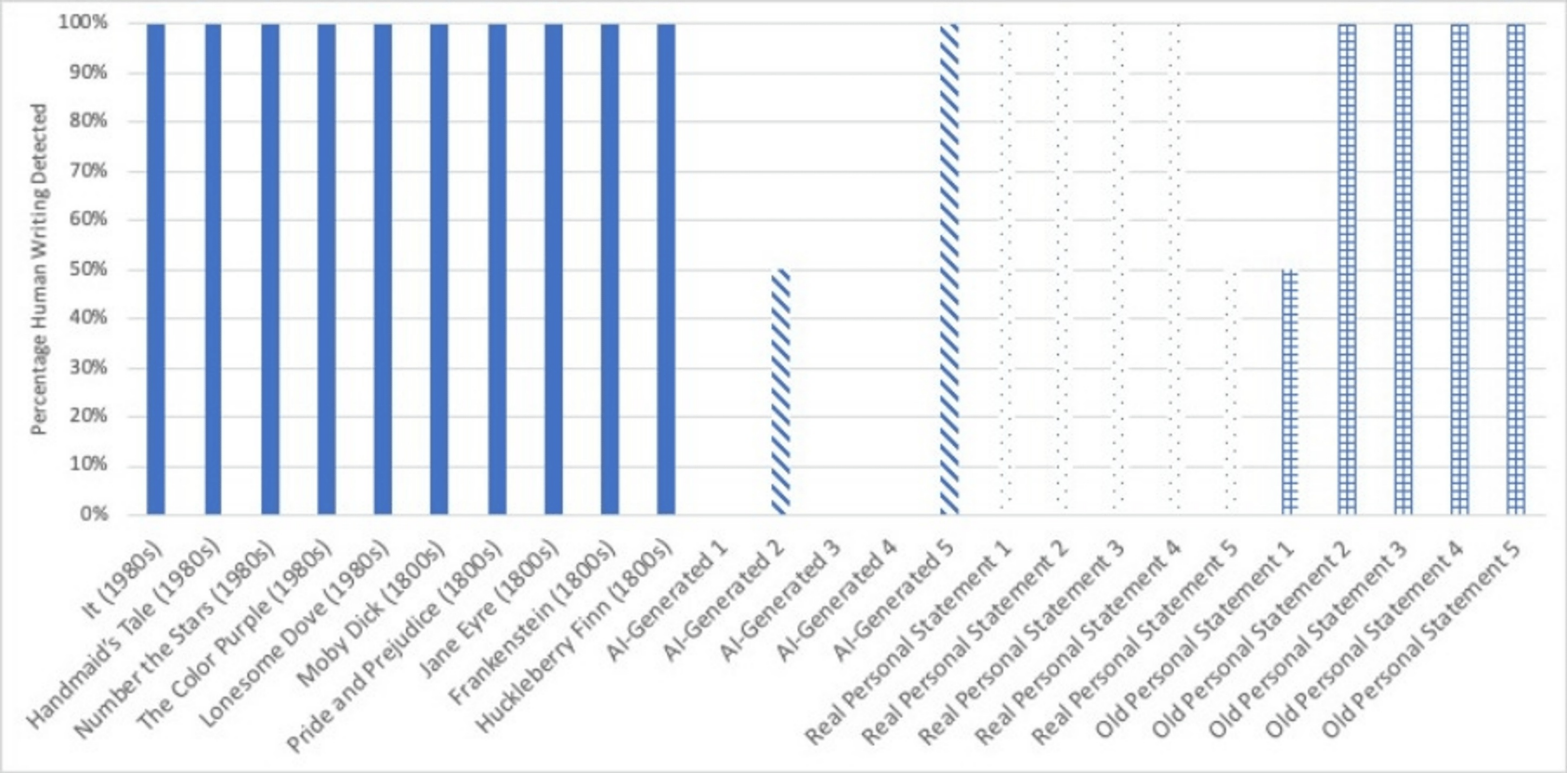
Undetectable AI results Undetectable AI results, percentage of writing samples detected as human in origin.

**Figure 3:**
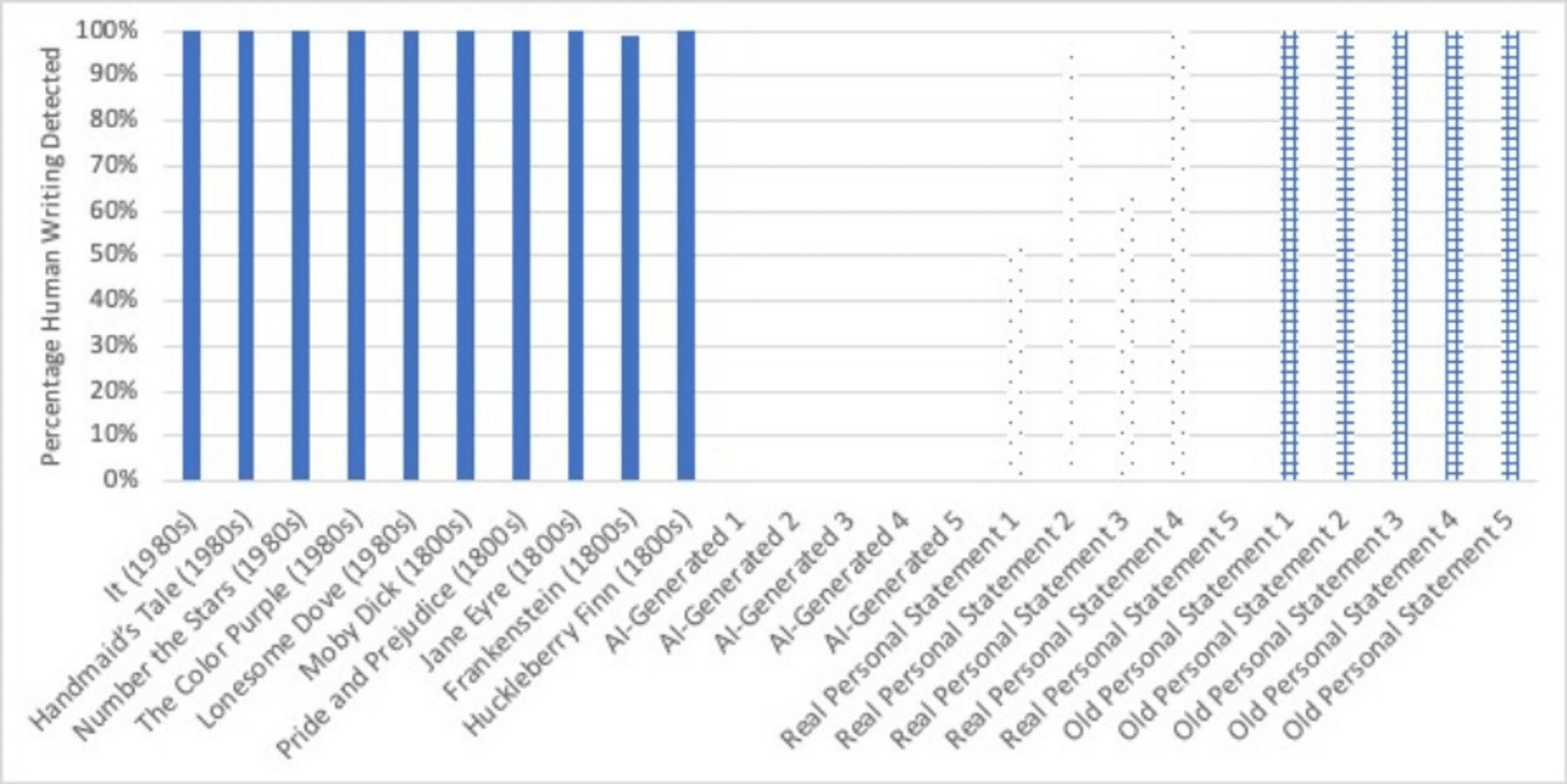
Winston AI results Winston AI results, percentage of writing samples detected as human in origin.

Figure 2 shows the Undetectable AI results, and Figure 3 shows the Winston AI results.

**Figure 4:**
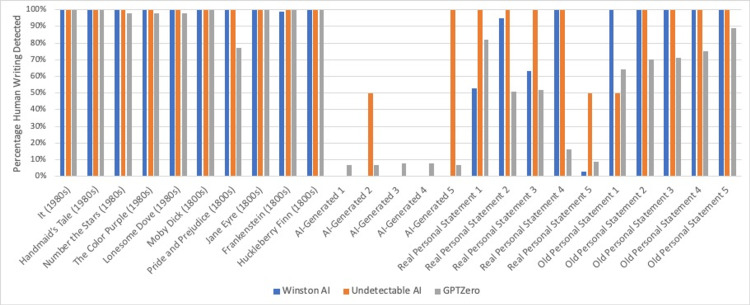
Winston AI vs Undetectable AI vs GPTZero Winston AI, Undetectable AI, and GPTZero percentage of human writing detected.

GPTZero's results were represented as a percentage, indicating the probability that the content was created by AI. Winston AI and Undetectable AI results also showed a probability, but this was the probability that the content was created by humans. In order to compare the data, we converted the GPTZero data to mirror the others. Figure 4 shows the results of different AI detectors, with GPTZero’s output converted from a percentage representing the probability that the content was created by AI to a percentage representing the probability that it was created by humans.

For the GPTZero detection system, writing samples from popular literary works pre-dating AI from the 1980s ranged from 0-2% likely to be created by AI, with 0% of the sentences likely generated by AI. For the Undetectable AI and Winston AI detection systems, the results were 100% human.

For the GPTZero detection system, writing samples from popular literary works pre-dating AI from the 1800s ranged from 0-23% likely to be produced by AI, with 0% of the sentences likely to be a product of AI. The Undetectable AI detection system yielded 100% human. Winston AI revealed almost all results as 100% human, with one sample 99% human.

For the GPTZero detection system, writing samples generated by AI were 92-93% likely to be entirely produced by AI, with 100% of the sentences likely generated by AI. Undetectable AI results ranged from 0-100% human, and Winston AI results were 0% human.

For the GPTZero detection system, writing samples directly from the 2023 Electronic Residency Application System cycle ranged from 18-91% likely to be created by AI, and 0-100% of the sentences were detected as likely to be generated by AI. Undetectable AI results were identified as 100% human, except for one personal statement, resulting as 50% human. Winston AI results ranged from 3-100% human.

For the GPTZero detection system, old writing samples that were submitted by real applicants in the Electronic Residency Application System prior to the launch of ChatGPT in 2022 ranged from 11-36% likely to be made by AI, with 0-37% of the sentences likely to be a product of AI. Undetectable AI results were 100% human, except for one writing sample. Winston AI results were 100% human.

## Discussion

In this study, we used popular and highly-ranked [9] AI detectors, including GPTZero, Undetectable AI, and Winston AI, to analyze five groups of writings with the goal of addressing the question of whether residency programs could detect undisclosed use of AI by applicants.

We found that classic novels (from both the 1800s and 1980s) had low rates of AI detection, which suggests that the AI detection software is working at least in regard to negative predictive value. AI-generated personal statements had high rates of detection, suggesting that these three AI detectors work effectively to detect AI-generated work. Real personal statements and old personal statements predating the technology had mixed results depending on the detector. For GPTZero and Winston AI, higher rates of AI use were seen in the real personal statements from 2023 compared to the old personal statements prior to the launch of ChatGPT. Undetectable AI called almost all of the real and old personal statements 100% human.

There are several possible explanations for these varied findings. AI large language models are taught using actual personal statements, which may bias the assessment of what is and is not AI-generated. Applicants may be using AI without disclosure. Applicants may not be using AI, and GPTZero, Undetectable AI, and Winston AI falsely label human writing as AI-generated writing, which represents a false-positive result. There is also a possibility that character count matters, and that uploading more or less content may impact the detection of AI-written text. It is clear here that not all AI detectors are the same, as GPTZero, Undetectable AI, and Winston AI had different results, as well as different ways of presenting them. Winston AI and Undetectable AI give a result that shows the probability that a writing sample appears to be created by a human as a percentage. While GPTZero also uses a percentage in its results, this percentage indicates the probability that a writing sample was created by AI.

It is unclear whether or not the real personal statements submitted by applicants in 2023 were crafted with any aid from ChatGPT, but it highlights the issue that we do not know who is utilizing ChatGPT in their writing, and we may not have a reliable way to detect unethical usage. None of the AI detectors in this study have been validated in the detection of AI use in writing, and no validated AI detectors were found in the literature. A preliminary study showed that GPTZero yielded 80% accuracy at detecting AI-generated text and had a low (10%) false-positive and a high (35%) false-negative rate [10]. While encouraging, this data is from a small study in one discipline, which limits the generalizability. Winston AI has posted its own accuracy rate (99.74%) along with the study performed by its own team. While this study was well-powered with 10,000 texts from humans and many different and newer AI technologies, it was performed by their team, and Winston AI is a paid service [11]. Undetectable AI is not its own detector; rather, it compiles results from eight other detectors: GPTZero, OpenAI (San Francisco, CA, USA), Writer (Writer, Inc., San Francisco, CA, USA); Crossplag (Beaverton, OR, USA); Copyleaks (Stamford, CT, USA); Sapling (Sapling Intelligence, Inc., San Francisco, CA, USA); Content at Scale (Glendale, AZ, USA), and ZeroGPT (Staffelstein, Germany).

Further, GPTZero and Winston AI caution that students should not be penalized solely based on its findings [8,11]. It is conceivable that authors may query any one of these AI detectors to analyze their document and make amendments to change the likelihood of AI use detection. Undetectable AI even offers a ‘humanize’ option to help the writer change their text to evade AI detectors. All of these variables must be considered when crafting academic policies regarding the use of AI.

Different organizations have varying stances on AI use. The *New England Journal of Medicine (NEJM) AI* will allow its use provided the authors take responsibility for content and acknowledge the use, with the caveat that AI itself cannot be listed as an author [1]. The *Journal of the American Medical Association* (JAMA) states, "only humans can be authors" [12,13]. When queried, ChatGPT itself responded that it cannot be an author in a medical journal [12].

The expectation is that a personal statement is just that, and represents original work by the applicant. Submission of a statement where the majority was written substantially by someone (or something) else is plagiarism, which is “to steal and pass off (the ideas or words of another) as one’s own” or “use (another’s production) without crediting the source” [14]. To complicate matters, the use of unvalidated tools by residency programs to detect AI use could adversely affect honest applicants who are submitting original work that could be flagged as written or assisted by AI.

While AI software has the potential to level the playing field for those who lack access to expensive writing services or writing coaches, the lack of disclosure of such use may worsen an already existing disparity. False positives, or the detection of AI where it was not used, can have negative implications for students not using the technology. False negatives, or the failure to correctly identify AI-generated work, can reward dishonest students who are not disclosing AI use in their work. These biases can wreak havoc on the academic environment if AI detection technology is utilized unchecked. AI is a tool that can enhance human intelligence, but not replace it. Universities and educational centers around the world are struggling to define the proper use of AI within their institutions. Many schools in New York City, Los Angeles, and Baltimore have already moved to ban ChatGPT [15], and Australian universities have posted rules characterizing the use of AI as cheating and moved towards pen-and-paper assignments [16].

We acknowledge that undisclosed authors or co-authors have been a potential issue in all forms of writing for centuries; however, the easy access to and seemingly innocuous use of AI chatbots to assist in writing make this issue much more pressing to address in modern society, especially in residency applications.

While we highlight some of the concerns of AI use in graduate medical education, there are beneficial ways to incorporate the software as well. Using resources such as ChatGPT can help students with understanding their curricula, provide options and ideas that can help accelerate learning, and help student development in core areas [17-21]. It can also function as a free editing service, reducing financial barriers to application supplementation [22]. We do not wish to condemn or support AI use in the residency selection process, merely propose adhering to guidelines for all non-author contributions to maintain transparency and justice.

This study has several strengths, including its qualitative approach and transparency. Analysis of writing samples is complex, and not easily defined, nor quantified in a meaningful way. Our study has some important limitations as well, including small sample size, the investigators being blinded to AI usage in our pool of real applicant submissions, and the use of only three of the available AI-detection software. Undetectable AI and Winston AI were chosen for their high rankings amongst top AI detectors [9]. GPTZero was chosen because it was created by OpenAI, the creator of ChatGPT, which is the most commonly used AI chatbot [23]. 

Currently, there is little guidance on the amount of AI technology that may be incorporated into a residency application. Use of AI to ease so-called writer's block may be acceptable, while asking an AI chatbot to write a 700-word personal statement as described in our Methods section may not be. Using ChatGPT or any AI technology to write a personal statement without disclosure, regardless of the amount of its contribution, seems to walk a dangerous line toward plagiarism, fraud, and academic misconduct.

We are not condemning the use of AI as a writing assistant; rather, we condemn the lack of disclosure. Such academic misconduct has been associated with dishonesty in the workplace, which is particularly concerning in the field of medicine [24,25]. We believe that submission of an AI-generated personal statement (in part or in whole) without disclosure of an outside source meets this definition. Others have identified this rising issue in medical education, and similarly found GPTZero had high detectability of AI-generated personal statements, and that AI-generated samples had longer sentences, lower readability, and complex vocabulary [26]. Winston AI and Undetectable AI were not previously studied in the literature, and were included in this study since they are widely used, highly rated, and provide additional options for AI detection [9].

With regard to the use of AI assistance in writing, we believe that most would agree that the principle of transparency must be adhered to in order to protect the integrity of our systems and organizations. Acknowledgement of references and proper use of quotations when writing in an official capacity is expected. Acknowledgement of co-authorship is mandatory when submitting a writing sample as your own, and a lack of such disclosure is improper. If an author’s contribution is more than half of the work required to complete the writing, they are considered the lead author. We believe that the use of AI assistance in the writing of a personal statement should be disclosed in accordance with the AMA and ICMJE. It should be made clear that failure to make this disclosure is sufficient for disqualification. Even with disclosure, the dynamic nature of this technology and developing detectors raises the question: will residency programs be able to effectively distinguish papers with AI components?

Available studies suggest that AI writing can be indistinguishable from human writing without the use of AI detectors [5]. In our study, AI detectors identified AI writing, but also misinterpreted human writing as AI-derived. This false positive identification does the honest human writer a disservice. This shortcoming illustrates that we need further development and testing within AI detectors and training in their use and limitations.

The role of AI large language models in the preparation (or writing) of personal statements for graduate medical education applications remains unclear. The potential to worsen disparity in the application process cannot be ignored. A transparency statement should be required with applicant submissions to enforce professional and ethical standards in our vocation and maintain equity in the residency admissions process. In order to address this concern and level the playing field, we suggest that applicants sign an attestation statement describing any use of AI in the creation of their personal statements. We encourage the requirement of a transparency statement as part of the personal statement since we are professionals held to a high standard in our field, and to uphold equity in the residency application process.

## Conclusions

Residency programs may be able to detect AI use in personal statements using AI-detection tools such as GPTZero, Undetectable AI, and Winston AI. Clear guidelines regarding authorship and AI assistance must be developed in order to maintain the integrity of student submissions. An attestation statement regarding the use of AI assistance in residency applications is an essential first step.
